# *Toxoplasma gondii* Me49 and NED strains arrest host cell cycle progression and alter chromosome segregation in a strain-independent manner

**DOI:** 10.3389/fmicb.2024.1336267

**Published:** 2024-02-21

**Authors:** Lisbeth Rojas-Barón, Carlos Hermosilla, Anja Taubert, Zahady D. Velásquez

**Affiliations:** Institute of Parasitology, Biomedical Research Center Seltersberg, Justus Liebig University Giessen, Giessen, Germany

**Keywords:** *Toxoplasma gondii*, haplotypes, Me49 strain, NED strain, cell cycle arrest, cell cycle dysregulation

## Abstract

*Toxoplasma gondii* is an obligate intracellular parasite that modulates a broad range of host cell functions to guarantee its intracellular development and replication. *T. gondii* includes three classical clonal lineages exhibiting different degrees of virulence. Regarding the genetic diversity of *T. gondii* circulating in Europe, type II strains and, to a lesser extent, type III strains are the dominant populations, both in humans and animals. Infections with the type I strain led to widespread parasite dissemination and death in mice, while type III is considered avirulent. Previously, we demonstrated that primary endothelial cells infected with the *T. gondii* RH strain (haplotype I) were arrested in the G2/M-phase transition, triggering cytokinesis failure and chromosome missegregation. Since *T. gondii* haplotypes differ in their virulence, we here studied whether *T. gondii*-driven host cell cycle perturbation is strain-dependent. Primary endothelial cells were infected with *T. gondii* Me49 (type II strain) or NED (type III strain), and their growth kinetics were compared up to cell lysis (6–30 h p. i.). In this study, only slight differences in the onset of full proliferation were observed, and developmental data in principle matched those of the RH strain. FACS-based DNA quantification to estimate cell proportions experiencing different cell cycle phases (G0/1-, S-, and G2/M-phase) revealed that Me49 and NED strains both arrested the host cell cycle in the S-phase. Cyclins A2 and B1 as key molecules of S- and M-phase were not changed by Me49 infection, while NED infection induced cyclin B1 upregulation. To analyze parasite-driven alterations during mitosis, we demonstrated that both Me49 and NED infections led to impaired host cellular chromosome segregation and irregular centriole overduplication. Moreover, in line with the RH strain, both strains boosted the proportion of binucleated cells within infected endothelial cell layers, thereby indicating enhanced cytokinesis failure. Taken together, we demonstrate that all parasite-driven host cell cycle arrest, chromosome missegregation, and binucleated phenotypes are *T. gondii*-specific but strain independent.

## 1 Introduction

*Toxoplasma gondii* is a major zoonotic obligate intracellular apicomplexan parasite and the etiological agent of toxoplasmosis, which may cause harmful effects mainly in pregnant and immunocompromised hosts. *T. gondii* modulates a broad range of host cell functions to guarantee its intracellular development and replication (Velásquez et al., 2019; Fernández-Escobar et al., 2022). As a polyxenous and cosmopolitan zoonotic parasite, *T. gondii* can infect all warm-blooded animals as intermediate hosts (humans, domestic and wild mammals, and birds) and domestic and wild felines as definitive hosts (Gubbels et al., 2008; White and Suvorova, 2018). Hence, its life cycle is a complex transmission process, and detailed molecular knowledge is of importance not only for public health but also for the livestock industry and wildlife management programs (Calero-Bernal et al., 2022).

Worldwide studies have shown that *T. gondii* possesses significant genetic and phenotypic diversity; at present, three main lineages (types I–III) are described, which vary in virulence and mortality for laboratory mice (Fernández-Escobar et al., 2021). Concerning the genetic diversity of *T. gondii* strains circulating in Europe, type II strains and, to a lesser extent, type III strains are the dominant populations, both in humans and animals (Khan et al., 2007; Lorenzi et al., 2016; Fernández-Escobar et al., 2022). The *T. gondii* type I strain is classified as highly virulent, leading to widespread parasite dissemination and lethal infection in mice (100% cumulative mortality). In contrast, mouse mortality and tachyzoite dissemination induced by type II or III strains are considerably lower (30%), with type III strains generally being considered avirulent for mice (Sibley and Boothroyd, 1992; Su et al., 2002; Dardé et al., 2014; Calero-Bernal et al., 2022). The main representatives for each lineage are *T. gondii* RH and GT1 for haplotype I, PRU and Me49 for haplotype II, and CEP and NED for haplotype III (Wu et al., 2022). However, the most studied strain in *in vitro* research is the *T. gondii* RH strain, while the NED strain is commonly used in murine *in vivo* models (Croken et al., 2014; Wang and Sibley, 2020).

It is well known that apicomplexan coccidian parasites extensively modulate their host cells to guarantee successful intracellular development and proliferation. As such, these parasites were reported to affect numerous host cellular processes, such as apoptosis, autophagy, cytoskeleton, metabolism, immune reactions, and cell cycle (Alberts et al., 2007; Dardé et al., 2014; Velásquez et al., 2019). Referring to the latter, the *T. gondii* RH strain arrested the host cell cycle in S-phase or at G2/M-phase transition, which was accompanied by a binucleated host cell phenotype and cytokinesis failure (Molestina et al., 2008; Velásquez et al., 2019). Moreover, RH strain-infected primary endothelial host cells showed supernumerary centrosomes in mitotic spindles, a displacement of single chromosomes from the equatorial plane, and dramatic chromosome missegregation errors (Velásquez et al., 2019). In mammals, the cell cycle is tightly regulated by several cell cycle-dependent cyclins and cyclin-dependent protein kinases (CDKs) that control cell cycle progression from G0/1 to M-phase (Alberts et al., 2007). During cell cycle, the formation of centrioles, the mitotic spindle, and the arrangement of chromosomes are also highly controlled to guarantee the correct genetic information to be inherited by daughter cells (Miettinen et al., 2019). Even tiny errors in these cellular processes might result in genome/chromosome instability or even cell death (Alberts et al., 2007). Former data on *T. gondii* RH infections stated cell type-dependent variations since human foreskin fibroblasts (HFFs) were arrested at the G2-to-M-boundary, while human trophoblast cells, human dermal fibroblasts, and L6 rat myoblasts showed stasis in G2-phase (Brunet et al., 2008; Molestina et al., 2008; Kim et al., 2016). In these studies G2-phase arrest was linked to cyclin B1 downregulation, while other G2/M-phase checkpoint-related molecules, such as p53, p21, and CDK1, were not changed in expression (Brunet et al., 2008; Velásquez et al., 2019). We recently demonstrated that *T. gondii* RH strain-infected primary endothelial cells experienced aberrant mitosis with supernumerary centrosome (and centriole) formation, resulting in impaired cytokinesis (Velásquez et al., 2019). Given that these data referred to haplotype I-driven host cell modulation, we aimed to determine whether cell cycle-related *T. gondii*-driven effects were haplotype/strain-dependent. For direct comparison and to avoid cell type-driven effects, *in vitro* infections with *T. gondii* haplotypes II and III (i.e., Me49 and NED strain, respectively) were performed in the same bovine primary endothelial host cell (BUVEC) as previously used for RH strain.

## 2 Materials and methods

### 2.1 Primary bovine umbilical vein endothelial cell isolation and culture

Primary bovine umbilical vein endothelial cells BUVEC were isolated from umbilical veins obtained from calves born by *section caesarea* at the Justus Liebig University in Giessen, Germany. Umbilical cords were maintained at 4°C in sterile 0.9% HBSS-HEPES buffer (pH 7.4; Gibco, Grand Island, NY, United States) supplemented with 1% penicillin (500 U/mL; Sigma, St. Louis, MO, United States) and streptomycin (500 μg/mL; Sigma) for a maximum of 12 h before use. Isolation of endothelial cells was performed by using 0.025% collagenase type II (Worthington Biochemical Corporation) suspended in Pucks solution (Gibco) and infused into the lumen of ligated umbilical veins for 20 min at 37°C in a 5% CO_2_ atmosphere. After gently massaging umbilical veins, the cell suspension was collected in medium and supplemented with 1 mL of fetal calf serum (FCS; Gibco) to inactivate collagenase. After two washes (350 x *g*, 12 min, RT), cells were resuspended in complete endothelial cell growth medium (ECGM, PromoCell, supplemented with 10% FCS), plated in 25 cm^2^ tissue plastic culture flasks (Greiner), and incubated at 37°C and 5% CO_2_ atmosphere. BUVEC monolayers were cultured in modified ECGM medium [ECGM, diluted at 30% in M199 medium, supplemented with 5% FCS (Greiner) and 1% penicillin and streptomycin] with medium changes every 2–3 days. All biological isolates were used for *in vitro* experiments at a maximum of four passages, as previously described in Velásquez et al. (2019). Experiments on bovine primary endothelial cells and parasites were performed following the permission of the Institute of Parasitology to work with biological agents up to risk class 3** [allowance according to §16 BiostoffVO, Az. GI 000056837, approved by the regional commission of Giessen (Regierungspräsidium Gieβen)], the Institutional Ethics Commission of Justus Liebig University of Giessen (Germany), and under the current European Animal Welfare Legislation: ART13TFEU.

### 2.2 Parasite maintenance

Tachyzoites of *T. gondii* Me49 and NED strains were maintained by serial passages in MARC-145 (African green monkey kidney epithelial cells) using DMEM medium (D6429, Sigma) supplemented with 5% FCS, 1% penicillin (500 U/mL; Sigma St. Louis, MO, United States), and streptomycin (500 μg/mL; Sigma). The number of passages for MARC-145 cells and *T. gondii* tachyzoites was controlled to compare our previous results on RH strains with those described here (Velásquez et al., 2019). *T. gondii* tachyzoites were obtained by monolayer scraping and centrifugation (400 x *g,* 1 min) to remove cell debris. A second centrifugation step was performed to sediment the parasites at 800 x *g* for 12 min. Tachyzoites were counted in a Neubauer chamber, suspended in a modified ECGM medium, and used for BUVEC infections.

### 2.3 Kinetics of *Toxoplasma gondii* infections

Confluent BUVEC layers (*n* = 3) were infected with *T. gondii* Me49 or NED tachyzoites (MOI 1:2) and incubated at 37°C in a 5% CO_2_ atmosphere. By counting the number of tachyzoites per parasitophorous vacuole (PV) every 6 h up to 30 h p. i., parasite infection kinetics in the same primary endothelial cell isolates were analyzed to estimate Me49- or NED-specific division cycles. Therefore, cells were fixed at each time point with 4% paraformaldehyde for 15 min at room temperature (RT) and then washed three times with 1X PBS buffer (137 mM sodium chloride, 2.7 mM potassium chloride, and 12 mM total phosphate in the form of hydrogen phosphate and dihydrogen phosphate). All fixed cells were stored at 4°C until further use. To assess parasite development in endothelial host cells, cell nuclei, and tachyzoites were labeled with 4′,6′-diamidin-2-phenyllindol (DAPI) and a specific *T. gondii* antibody, respectively (Table 1), allowing us to count the total number of tachyzoites/PV formed during infection.

**Table 1:** Primary and secondary antibodies used in the current study.

Antigen	Company	Cat. number	Origin/reactivity	Dilution
**Primary antibodies**				
*T. gondii*	Thermo Fisher	PA1-7256	Goat	1:100
γ-Tubulin	Abcam	Ab1795030	Rabbit	1:100
Vinculin	Santa Cruz	sc-73614	Mouse	1:1000
Cyclin A2	Abcam	Ab38	Mouse	1:1000
Cyclin B1	Abcam	Ab32053	Rabbit	1:3000

Antigen/Conjugate	Company	Cat. number	Host/target	Dilution
**Secondary antibodies**				
Alexa Fluor 594	Thermo Fisher	A-21468	Goat	1:500
Alexa Fluor 647	Thermo Fisher	A-21244	Rabbit	1:500
Goat anti-mouse IgG Peroxidase conjugated	Pierce	31430	Goat/mouse	1:40,000
Goat anti-rabbit IgG Peroxidase conjugated	Pierce	31460	Goat/rabbit	1:40,000

### 2.4 Immunofluorescence assays

Three BUVEC isolates were seeded in 12-well plates with coverslips precoated with fibronectin (1:400, Sigma-Aldrich, F1141-2MG) and infected either with *T. gondii* Me49 or NED tachyzoites at sub-confluency (MOI 1:2). At 24 h p. i., all samples were fixed in 4% paraformaldehyde (15 min, RT) and washed three times in sterile PBS. The samples were incubated in a blocking/permeabilization solution (PBS with 3% BSA and 0.3% Triton X-100) for 1 h at RT. Thereafter, they were incubated in primary antibody solutions (Table 1) at 4°C in a humidified chamber overnight. The samples were then washed three times with 1X PBS and incubated in secondary antibody solutions (Table 1) for 30 min at RT and darkness. Host cell nuclei were labeled with DAPI present in the mounting medium solution (Fluoromount G-DAPI, Thermo Fisher, cat. Number 495952). The samples were analyzed with ReScan Confocal instrumentation (RCM 1.1 Visible, Confocal.nl) combined with a Nikon Eclipse Ti2-A inverted microscope.

### 2.5 Protein extraction

Six BUVEC isolates were infected with either *T. gondii* Me49 or NED tachyzoites (MOI 1:2). At 24 h p. i., cells were washed with 1X PBS buffer, detached from the plate using trypsin/EDTA solution [0.25% (w/v) Trypsin; 0.53 mM EDTA, 37°C, 5 min], and pelleted (400 *x g*, 5 min). The cell pellet was washed with 1X PBS buffer and resuspended in RIPA buffer (50 mM Tris–HCl, pH 7.4; 1% NP-40; 0.5% Na-deoxycholate; 0.1% SDS; 150 mM NaCl; 2 mM EDTA; 50 mM NaF; all Roth) supplemented with a protease inhibitor cocktail (Sigma-Aldrich), 1 mM sodium orthovanadate tyrosine phosphatase inhibitor (Abcam, ab120386), and 1 mM phenylmethylsulphonyl fluoride, a serine protease inhibitor (Abcam, ab141032). Protein extracts were sonicated for five cycles of 20 s sonication and 20 s resting and then centrifuged (10,000 x *g*, 10 min, 4°C) to sediment intact cells, membranes, and nuclei. The supernatants were analyzed for protein content via BCA protein assay (Pierce BCA Protein Assay Kit, Thermo Scientific, cat. Number 23225) following the manufacturer’s instructions. Sample analysis was performed on a Varioskan plate reader, measuring the absorbance at 562 nm.

### 2.6 SDS-PAGE and immunoblotting

Protein extracts were diluted in loading buffer with 6 M urea (10% SDS, 12.5% 2-mercaptoethanol, 25% glycerol, 150 mM Tris–HCl pH 6.8) and boiled at 95°C for 5 min. Samples (40 μg protein/slot) were loaded on 12% polyacrylamide gels and subjected to SDS-PAGE electrophoresis (100 V; approx. 1.5 h; Bio-Rad). Proteins were transferred to PVDF membranes (Millipore) (300 mA, 2 h) in a wet-tank transfer system and then blocked for 1 h at RT [3% BSA in TBS buffer (50 mM Tris–HCl, 150 mM NaCl; pH 7.6)]. Afterwards, proteins on membranes were incubated in primary antibodies (4°C, overnight) directed against cyclin A2, cyclin B1, and vinculin (Table 1) and diluted in blocking solution (TBS buffer, 0.1% Tween-20, 3% BSA). Vinculin detection was used as a loading control for sample normalization. After primary antibody probing, membranes were washed three times with TBS-Tween (0.1%) and incubated in secondary antibody solutions (Table 1) for 30 min at RT. After three washings (TBS-Tween 0.1%), protein detection was performed using a chemiluminescence detection system (ECL Prime, Amersham). Images were taken using the INTAS Science Imaging Instrument and the INTAS ChemoStar Imager software. A protein ladder was used to estimate protein sizes (PageRuler Plus Prestained Protein Ladder, Thermo Fisher Scientific). Protein band intensities were analyzed using the Fiji Gel Analyzer plugin (Schindelin et al., 2012).

### 2.7 Flow cytometry-based analysis of cell cycle phases

For flow cytometry (FACS)-based analysis of the cellular DNA content, non-infected and infected host cell layers (*n* = 6) were washed with 1X PBS buffer and detached from the plate by trypsin/EDTA (0.25%) treatments at 37°C for 5 min. Then, cells were pelleted at 400 x *g* for 5 min. The cell pellet was resuspended, washed in 1X PBS buffer, and centrifuged at 400 x *g* for 5 min. Thereafter, cells were fixed with ice-cold absolute ethanol for 3 min at −20°C and pelleted again (400 x *g,* 5 min). Samples were stained with the commercial FxCycle PI/RNAse kit following the manufacturer’s instructions (Thermo Fisher, F10797). The samples were analyzed by an Accuri C6 Plus Flow Cytometer analyzer (Becton-Dickinson, Heidelberg, Germany) applying 535/5 nm excitation and emission collected in a 617/20 band-pass. The cells were gated according to their size and granularity. Data analysis was performed via FlowJo LLC software (Ashland, OR).

### 2.8 Image acquisition and reconstruction

Fluorescence images were acquired with a ReScan Confocal instrumentation (RCM 1.1 Visible, Confocal, The Netherlands) equipped with a fixed 50 μm pinhole and combined with a Nikon Eclipse Ti2-A inverted microscope with a motorized Z-stage (DI1500, Nikon). The RCM unit was connected to a Toptica CLE laser with the following excitation modes: 405/488/561/640 nm. Images were taken via an sCMOS camera (PCO edge) using a CFI Plan Apochromat X60 lambda-immersion oil objective (NA 1.4/0.13; Nikon). The instrument was operated by the NIS-Elements software (version 5.11). Images were acquired via a z-stack optical series with a step size of 0.1 microns to cover all structures of interest. To estimate both the total number of cells and the number of binucleated cells present in one cell layer, all images were first segmented using the Otsu thresholding algorithm. Identical brightness and contrast conditions were applied for each data set within one experiment. The total number of cells was obtained using the Fiji plugin “Analyzes particles” with a size of 10 μm (Schindelin et al., 2012).

#### 2.8.1 Mitosis quantification

The percentage of cells that underwent mitosis was calculated by normalizing it with the total number of cells in each field of view. Mitosis was evaluated from the prophase until the telophase. Cytokinesis was evaluated in terms of the percentage of binucleated cells. The prophase was defined as the chromosome condensation in the nuclear region using DAPI staining of the chromosomes. Metaphase cells were those with all chromosomes in the equatorial line, and anaphase cells were when chromosomes left the central plane to migrate into each mitosis pole. Cells in anaphase with a DAPI-positive signal in between were identified as chromosome bridges. Finally, cells in telophase were counted when chromosomes had completely migrated to each mitosis pole.

### 2.9 Statistical analysis

The data were expressed as the mean ± SD of independent experiments. For cell number- and FACS-based experiments, one-way analysis of variance (non-parametric ANOVA) with Kruskal-Wallis post-test was performed using GraphPad Prism 9.3.1 software, applying a significance level of 5%. For immunoblot-based analyses, unpaired two-tailed *t-*tests were performed comparing controls vs. infected cells, with a 95% confidence interval. All graphs and statistical analyses were performed using GraphPad Prism 9 software.

## 3 Results

### 3.1 *Toxoplasma gondii* Me49 and NED intracellular development show comparable kinetics in primary endothelial cells

The current aim was to evaluate if variable *T. gondii* strains may differentially affect the host cell cycle during tachyzoite intracellular development. Given that diverse *T. gondii* haplotypes bear different virulence in the murine system, which may be linked to varying speed in development and cell lysis, we first analyzed the developmental characteristics of Me49 and NED strains (haplotypes II and III, respectively) in primary BUVEC layers. The rationale to choose a primary bovine endothelial cell type as host cells was: (i) to avoid immortalization-driven effects on cell cycle regulation as commonly reported for permanent tumor-based cell lines, (ii) to be as close as possible to the *in vivo* scenario, and (iii) to perform current studies in exactly the same cell type as reported before for the *T. gondii* RH strain [haplotype I, (Velásquez et al., 2019)] thereby allowing for direct data comparison. Therefore, BUVEC and MARC-145 passages, as well as *T. gondii* tachyzoites, were carefully controlled.

**Figure 1:**
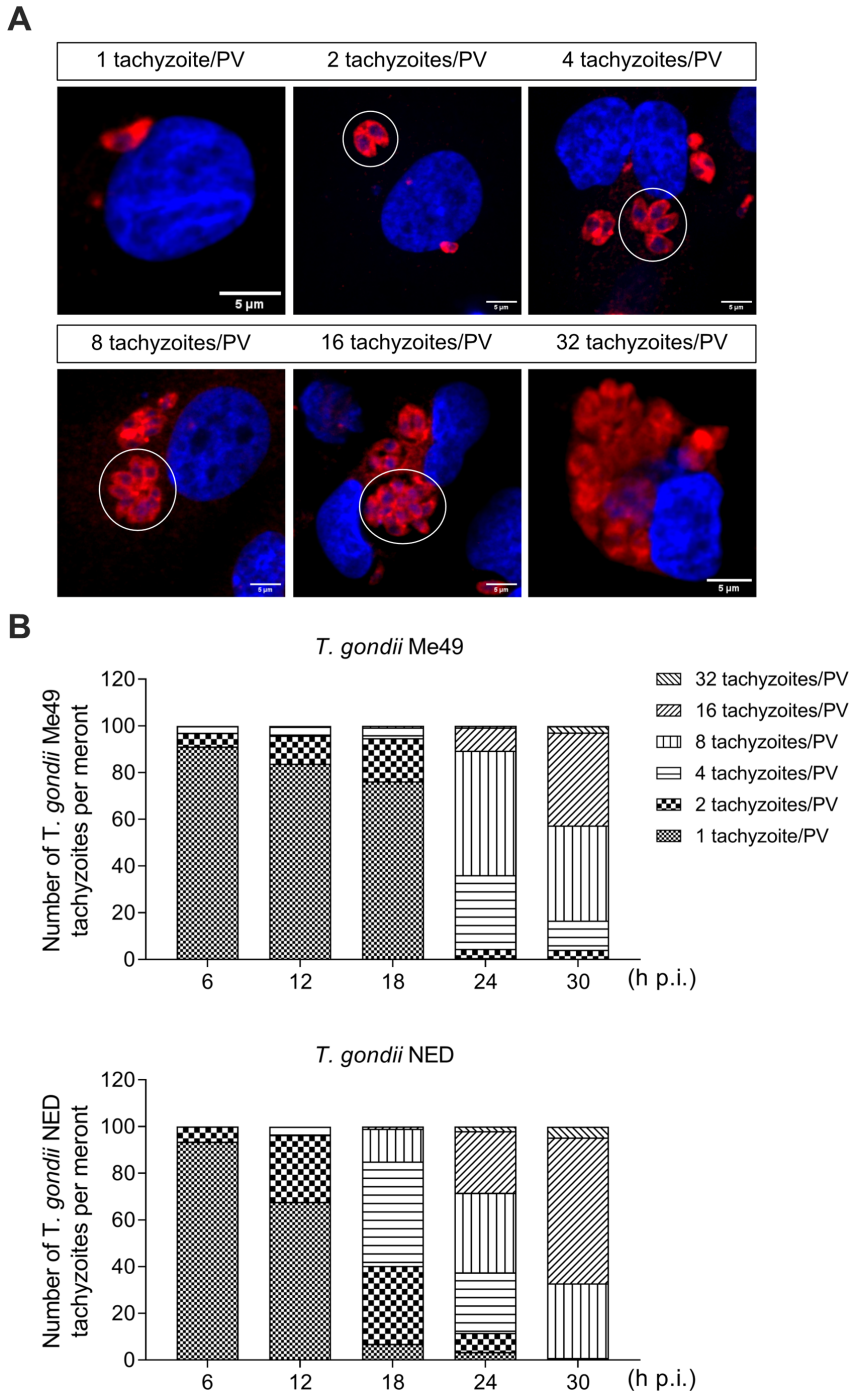
*Toxoplasma gondii* Me49 and NED tachyzoite replication kinetics in primary bovine umbilical vein endothelial cells (BUVEC). BUVEC isolates *(n* = 3) were simultaneously infected at subconfluence with either Me49 or NED tachyzoites (MOI 1:2) and monitored for parasite proliferation by analyzing the numbers of tachyzoites/parasitophorous vacuole (PV, white circle) from 6 to 30 h p. i. **(A)** Representative illustration of different stages of proliferation. Host cell nuclei were stained by DAPI (blue) and tachyzoites by *T. gondii*-specific antibodies (red). **(B)** Estimation of the proliferation status of Me49 and NED strains at 6, 12, 18, 24 and 32 h p. i. The scale bar represents 5 μm.

To study the kinetics of parasite development, identical BUVEC isolates were simultaneously infected with tachyzoites of *T. gondii* Me49 and NED strains, and the total intracellular development of each strain was thoroughly assessed by counting the number of tachyzoites per PV every 6 h from 6 to 30 h p. i. (Figure 1). Given that the host cells used in the current study were of primary origin and therefore not immortalized, three biological isolates at a maximum of four passages were used to avoid potential age-dependent changes in cell division times and further to ensure preservation of the endothelial phenotype. All host cells were seeded and infected at the same time, using the same batch of tachyzoites from each strain. A rosette was defined as when the PV contained 32 tachyzoites. All samples were analyzed before 32 h p. i. to avoid cell lysis-driven artifacts.

When analyzing in total 895 and 770 single infected host cells for NED and Me49 strains, respectively, at 24 h p. i., the overall infection rates differed moderately but not substantially (Me49: 81.9 ± 3.1%; NED: 70.1 ± 7.7%). The onset of tachyzoite division revealed equal in both strains at 6 h p. i. 2.5% of infected BUVEC showed two tachyzoites per PV for both strains (Figures 1A,B). However, at 12 and 18 h p. i., the NED strain proceeded slightly faster in development than the ME49 strain, since a higher proportion of PV contained four and eight tachyzoites (ME49: 1.3 and 5.7%, respectively; NED: 2 and 16.9%, respectively) (Figures 1A,B). Nevertheless, towards 30 h p. i., the NED strain caught up in development, thus resulting in a comparable proportion of PV with 32 tachyzoites (ME49: 1.4%; NED: 1%) (Figures 1A,B). Of note, BUVEC lysis started at comparable time points at 33 h p. i., thereby denying any relevant differences in the *in vitro* virulence of these two *T. gondii* strains. Based on these overall findings, it appeared eligible to perform cell cycle-related experiments on both strains at the same time points after parasite infection.

### 3.2 *Toxoplasma gondii* Me49 and NED tachyzoite infections both induce binucleated host cells and affect centriole formation

**Figure 2:**
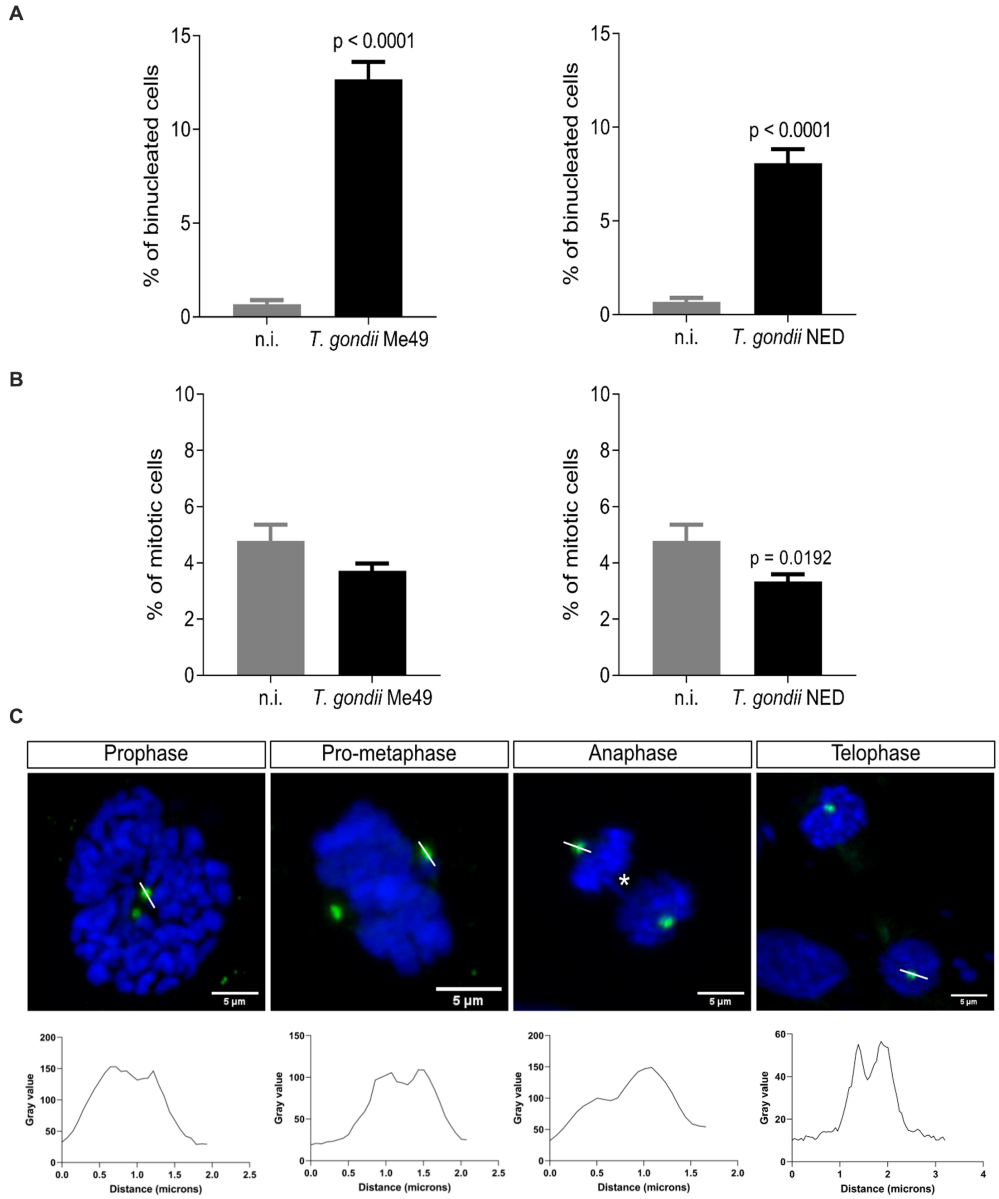
*Toxoplasma gondii* Me49 and NED infections induce a binucleated phenotype and centriole overduplication in infected host cells. Subconfluent BUVEC isolates (*n* = 3) were infected with either Me49 or NED tachyzoites at an MOI 1:2. After 24 h p. i., samples were stained for DNA/chromosomes (DAPI, blue) and centrioles (γ-tubulin, green). **(A)** Percentages of host cells with a binucleated phenotype. **(B)** Mitotic rate (cells undergoing mitosis/total number of cells) of *T. gondii*-infected BUVEC and control cells. **(C)** Exemplary illustration of the main aberrant mitotic structures observed for both Me49 and NED strain infections. Additionally, the intensity of centriole-related signals was assessed and plotted as a graph showing intensity value vs. distance (white line). Yellow arrows: centriole overduplication; asterisks: chromosome bridge. The scale bar represents 5 μm.

It was previously described that *T. gondii* RH strain infection in BUVEC layers resulted in an enhanced proportion of bi/multi-nucleated host cells (thereby indicating cytokinesis failure) and an alteration of mitosis progression by inducing supernumerary centrosome formation and chromosome segregation errors (Molestina et al., 2008; Velásquez et al., 2019). In this study, we intended to study whether these findings also applied to other *T. gondii* haplotypes (i.e., Me49 and NED). Therefore, the number of bi/multi-nucleated host endothelial cells (i.e., with ≥ 2 nuclei per cell) was counted and normalized against the total number of cells present in the field of view. As depicted in Figure 2A, both Me49 and NED infections of BUVEC induced a significantly enhanced proportion of bi/multi-nucleated host cells (Me49: 12.7%; NED: 8.1%), while only 0.7% of non-infected cells revealed the binucleated phenotype. The mitotic rate was also analyzed by estimating the total number of cells in mitosis vs. the total number of cells in the field of view. The results showed that only the NED strain reduced statistically significant mitosis percentages (Figure 2B). To verify whether mitotic failures may be linked to inadequate mitotic spindle and centrosome formation, we additionally stained chromosomes with DAPI (blue) and centrosomes with γ-tubulin (green) (Figure 2C) and analyzed the different phases of mitosis in *T. gondii* Me49- and NED-infected BUVEC. The γ-tubulin staining was used as a centrosome marker to visualize the mitosis spindle localization. As expected, most of the mitotic cells displayed a normal mitosis progression with only two centrosome poles. However, approximately 10% of infected mitotic cells showed an altered chromosome arrangement (Figure 2C, prophase and metaphase). A low number of host cells displayed chromosome bridges between the two poles of the mitotic spindle (Figure 2C, asterisks, and Supplementary Video S2). Interestingly, the centrosomes seem to be composed of more than one spot (Figure 2C, and Supplementary Video S1). To corroborate this observation, we drew a line over the centrosomes in mitotic cells, and we plotted the information in a histogram. The results showed that in most of the cases, the histogram exhibited two brightness dots, suggesting a possible centriole duplication at each centrosome (Figure 2C, histogram plots, and Supplementary Video S1).

### 3.3 *Toxoplasma gondii* ME49 and NED infections both promote host cell cycle arrest in S-phase

**Figure 3:**
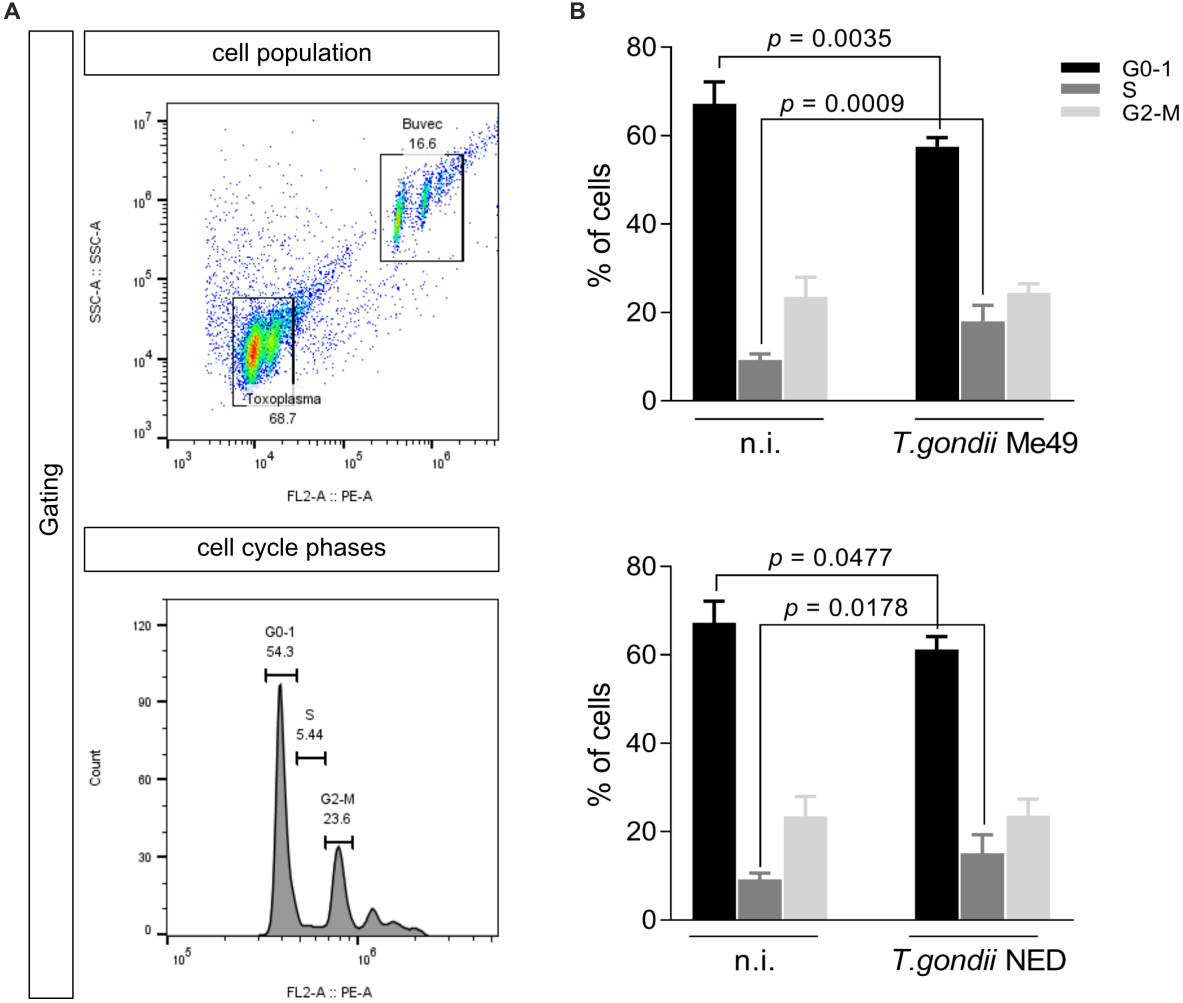
*Toxoplasma gondii* Me49 and NED infections arrest the host cell cycle in S-phase. Subconfluent BUVEC isolates (*n* = 6) were infected with either Me49 or NED tachyzoites at an MOI 1:2 and evaluated for DNA content at 24 h p. i. **(A)** Exemplary flowchart of FACS analysis showing the total number of host cells in G0-1 (one genomic DNA copy), G2- (two genomic copies), and S-phase (DNA synthesis, the cell population in between both phases). The number of cells positive for DNA staining (PI) was graphed as a histogram to obtain the total number of cells in each peak. **(B)** Percentages of Me49- and NED-infected BUVEC and control cells in each cell cycle phase were estimated via FACS-based DNA quantification. Bars represent the median ± SD.

To analyze whether *T. gondii* Me49 or NED strains cause dysregulation of the host cell cycle progression in BUVEC, a FACS-based analysis was performed to estimate total DNA content. The cell population was gated according to their size and granularity, and PI-based DNA staining was used to define distinct categories of cell cycle phases (G0/1, S, and G2/M) according to the DNA amount per cell (Figure 3A). In this classical method, the first DNA peak is assigned to G0/1-phase, the second peak represents cells in G2/M-phase, and the cell population between both peaks corresponds to cells in the S-phase (Figure 3A). Here, *T. gondii* Me49- and NED-infected cells both showed a significant decrease of cells in G0/1-phase when compared to non-infected cells. Simultaneously, the proportion of *T. gondii*-infected cells in the S-phase increased, thereby suggesting a parasite-driven host cell arrest in the S-phase (Figure 3B).

**Figure 4:**
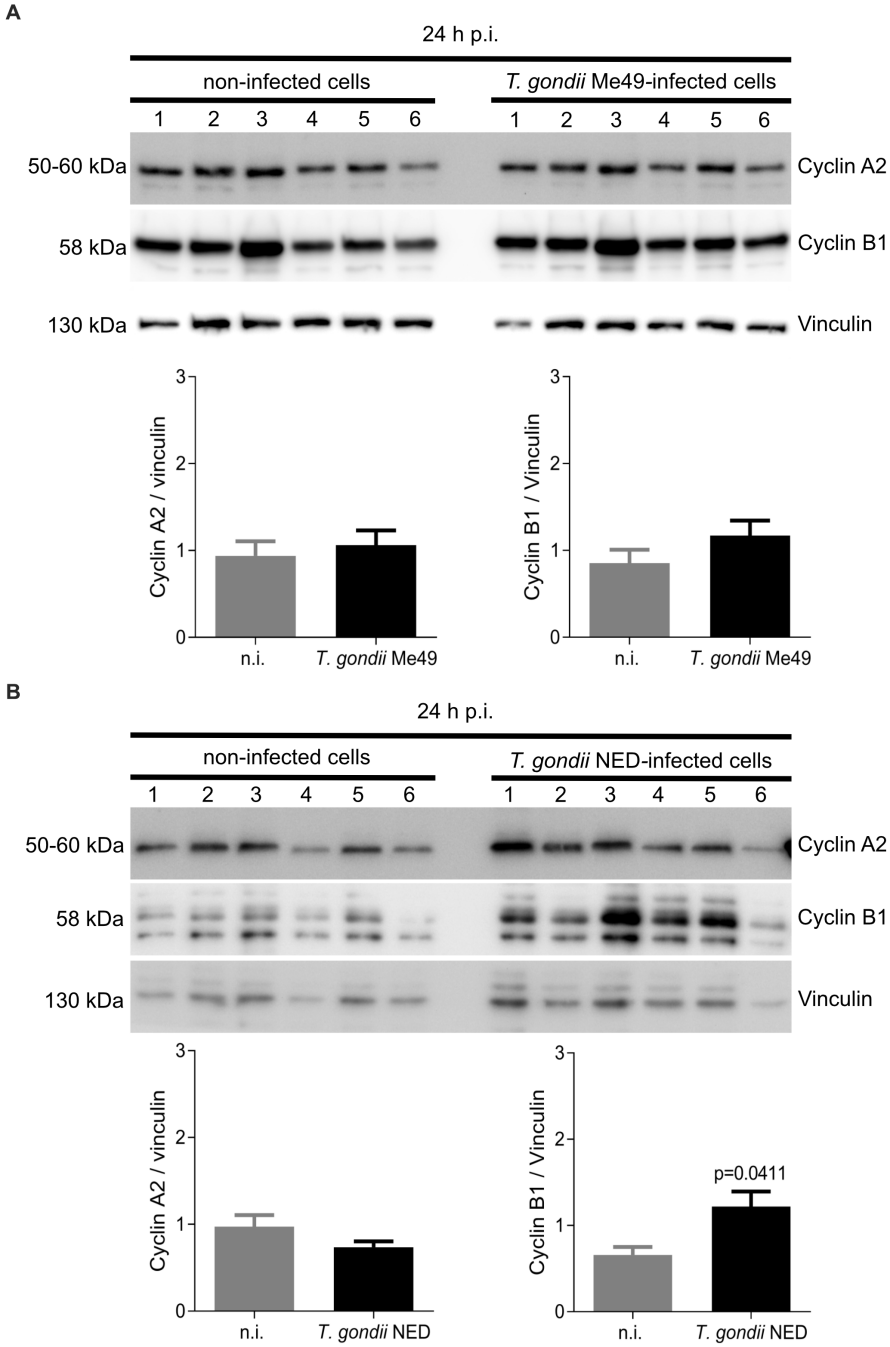
Cyclin A2 and cyclin B1 expression in *Toxoplasma gondii* Me49 and NED strain-infected BUVEC. Confluent BUVEC isolates (*n* = 6) were infected with either Me49 **(A)** or NED **(B)** tachyzoites. At 24 h p. i., protein extracts were analyzed by Western blotting to estimate cyclin A2 (indicative for S-phase) and cyclin B1 (mitosis marker) expression. Densities of protein signals were quantified and graphed as a relative ratio to vinculin expression (loading control). Bars represent the median ± SD of six biological replicates.

Since cellular DNA content-based analyses do not allow for discrimination between all single phases (such as G0/G1 or G2/M) and based on current data suggesting that *T. gondii*-infected host cells accumulate in the S-phase, we additionally analyzed the expression of cyclins A2 and B1 (Figure 4), which signify key regulatory proteins of S-phase control and of M-phase enter and progression, respectively. Western blotting analyses of six ME49- and NED-infected BUVEC isolates showed that only the NED strain induces cyclin B1 overexpression (Figure 4B). Cyclin A2 expression was not affected by any of the strains (Figures 4A,B).

## 4 Discussion

In a recent study, we demonstrated that *T. gondii* tachyzoites of haplotype I (RH strain) induced host cell cycle arrest, chromosome missegregation, multipolar spindle, and cytokinesis failure concomitant with an increased percentage of binucleated primary host endothelial cells *in vitro* (Velásquez et al., 2019). To assess eventual strain-driven effects, we compare our previous published data with infections using two other *T. gondii* haplotypes, II and III (Me49 and NED, respectively). We evaluated the host cell cycle control, progression, and mitosis after 24 h h p. i. with *T. gondii* tachyzoites Me49 and NED. To be able to compare our results with previously published data, the same host cell type was used and parasite strain passages were tightly controlled. Furthermore, we followed the same experimental approach used in Velásquez et al. (2019).

To our best knowledge, available data on *T. gondii*-driven host cell cycle modulation currently refer to haplotype I tachyzoites (RH strain) and indicate that this clonal lineage might control host cell cycle progression to ease its intracellular asexual development (Brunet et al., 2008; Molestina et al., 2008; Velásquez et al., 2019; Wong et al., 2020; Pierre-Louis et al., 2022). Since several typical and atypical clonal lineages of *T. gondii* occur worldwide and show variable pathogenicity (Dardé et al., 2014; Miller et al., 2023), we aimed to compare recent RH data (haplotype I) (Velásquez et al., 2019) with haplotypes II and III (Me49 and NED, respectively) by infecting the same host cell type. In our hands, the replication times of the ME49 and NED strains in BUVEC proved comparable to those of the RH strain, allowing us to use this primary cell culture system for comparative approaches. Our results showed that the NED strain reduced the mitosis rate and led to an overexpression of cycin B1, in contrast to the Me49 strain. This suggests that the NED strain modulates the host cell cycle throughout the mitosis checkpoint, while ME49 and RH appear to modulate whitin S-phase progression itself. Since neither *T. gondii* RH nor ME49 controls the mitosis checkpoint cyclin, even though both arrest the host cell cycle, *T. gondii* tachyzoite has evolved into several clonal lineages, with the most prevalent ones being types I, II, and III. While all lineages have the potential to infect both humans and animals, types I and II are more prevalent in immunocompromised or pregnant humans than in animals. Interestingly, type III primarily infects animals as opposed to people (Howe and Sibley, 1995). In our findings, the NED strain was the one to have distinct behavior about the cell cycle and mitosis control. Therefore, this slight modification may suggest that the NED strain evolved into a better-intermediated host adaptation, modifying the type of the host cell cycle and mitosis control. However, further experiments need to be done in order to answer this hypothesis.

Referring to parasite-mediated modulation of cell cycle progression, the current data showed that both NED and ME49 strains induced host cell cycle arrest in S-phase, but they differed in the control, thereby denying any haplotype-dependent reactions. Interestingly, S-phase arrest has been studied by other groups worldwide, suggesting that arrested cells were not able to incorporate new DNA molecules (Pierre-Louis et al., 2022). Given that *T. gondii* arrests the host cell cycle equally in primary, immortalized, and tumor cells, we could suggest that it is a parasite strategy that has been maintained throughout haplotype evolution (Brunet et al., 2008; Molestina et al., 2008; Kim et al., 2016; Velásquez et al., 2019; Pierre-Louis et al., 2022). The mitosis progression was shown to be modulated differently in NED- or ME49-infected cells compared to previous data published on the RH strain since infected cells displayed multipolar spindle formation (Velásquez et al., 2019). In this study, we showed that neither ME49 nor NED strains induce multipolar spindles. However, the centrosome poles seem to have more than one spot of γ-tubulin, suggesting possible centriole duplication. This phenotype was also observed in the RH strain infection, but it always came with a multipolar phenotype. Altogether, this suggests that *T. gondii* modulation of the centriole number can be independent of the strain type, but the multipolar spindle formation may only be induced by the RH strain.

During the process of chromosome segregation and mitotic spindle formation, several specific proteins are needed, such as histones and cohesins, to deliver structural support for chromosomes and to bind sister chromatids to ensure an equal distribution between daughter cells during cell division (Kline-Smith and Walczak, 2004; Mariño-Ramírez et al., 2005; Makrantoni and Marston, 2018). The loss or gain of chromosomes drives chromosomal errors and triggers intracellular pathways that arrest the cell cycle to either repair the damage or eliminate potential aneuploid cells by apoptotic death (Alberts et al., 2007; Levine and Holland, 2018). The same applies to irregular chromosome bridges, which were found here to be induced by *T. gondii* Me49 and NED strains and that have been correlated with DNA damage (Ganem and Pellman, 2012). As described by Pampalona et al. (2016), chromosome bridges are characteristic of tumor cells and are mainly observed in mid-late anaphase, eventually persisting throughout mitosis, but are atypical for the early G1 phase. Since the endothelial host cells used in the current study are of primary and non-tumoral origin and, consequently, should follow physiological cell cycle control, chromosome bridge-related findings should be associated with *T. gondii* infection. However, considering that chromosome bridging is better monitored by live-cell imaging, future experimentation will focus on this method to reliably correlate these findings with *T. gondii*-mediated effects.

In line with recent data on the *T. gondii* RH strain (Velásquez et al., 2019), we demonstrated that ME49 and NED infections of BUVEC also induced an increased percentage of binucleated cells, which directly correlates with enhanced cytokinesis failure. Cytokinesis represents the last pivotal step of cell division. Hence, cytokinesis includes cytoplasmic division finally giving rise to two daughter cells, even when some exceptions have been described in the early embryonic stages of the fruit fly model *Drosophila* (Schejter and Wieschaus, 1993; Kiseleva et al., 2001). In mammals, megakaryocytes (blood platelets), hepatocytes, and heart muscle cells perform nuclear division without cytokinesis, leading to a high proportion of multi-nucleated cells (Nagata et al., 1997; Ahuja et al., 2007; Alberts et al., 2007; Margall-Ducos et al., 2007; Lordier et al., 2008). However, the endothelium of vessels *in vivo* physiologically does not include binucleated phenotypes, therefore evidencing that our findings are directly correlated with *T. gondii* infections. Given that—with the current study—this phenotype is now described for haplotypes I–III *in vitro*, binucleated phenotypes and cytokinesis failure may be considered a specific hallmark of *T. gondii* tachyzoite replication. Whether this phenomenon is indeed not linked to strain virulence seems likely but should be tested *in vivo* for further clarification.

Taking all the data together, we can suggest that host cell cycle modulation, chromosome segregation, and cytokinesis failure are intrinsic mechanisms of *T. gondii* tachyzoite infection and are independent of the parasite haplotype or virulence.

## Data availability statement

The original contributions presented in the study are included in the article/Supplementary material, further inquiries can be directed to the corresponding author.

## Ethics statement

The experiments on bovine primary endothelial cells and parasites were performed following the permission of the Institute of Parasitology to work with biological agents up to risk class 3** [allowance according to §16 BiostoffVO, Az. GI 000056837, approved by the regional commission of Giessen (Regierungspräsidium Gieβen)], Institutional Ethics Commission of Justus Liebig University of Giessen (Germany), and under the current European Animal Welfare Legislation: ART13TFEU. The study was conducted in accordance with the local legislation and institutional requirements.

## Author contributions

LR-B: Conceptualization, Data curation, Formal analysis, Methodology, Software, Validation, Visualization, Writing – original draft, Writing – review & editing. CH: Funding acquisition, Resources, Writing – review & editing. AT: Conceptualization, Funding acquisition, Investigation, Resources, Supervision, Writing – review & editing. ZV: Conceptualization, Data curation, Formal analysis, Investigation, Methodology, Software, Supervision, Visualization, Writing – review & editing.
