# Supplementary material for: Toxoplasma gondii Me49 and NED strains arrest host cell cycle progression and alter chromosome segregation in a strain-independent manner
Source: Front Microbiol. 2024 Feb 21;15:1336267. doi: 10.3389/fmicb.2024.1336267 (PMC10915083; doi:10.3389/fmicb.2024.1336267)
Supplement: Supplementary file 3 [file Data_Sheet_1.pdf]

Supplementary Material

1. Supplementary Material captions

Supplementary video 1: *Toxoplasma gondii* Me49 and NED infections induce a centriole overduplication in infected host cells. 3D reconstruction of z-stack confocal images using the maximum intensity projection algorithm. The video shows a prometaphase with and overduplicated centrioles (green). Cells were stained for DNA/chromosomes (DAPI, blue) and centrioles ((γ-tubulin, green).

Supplementary video 2: *Toxoplasma gondii* Me49 and NED infections induce a centriole overduplication in infected host cells. 3D reconstruction of z-stack confocal image using the maximum intensity projection algorithm. The video shows an anaphase with overduplicated centrioles (green) and a bridge formed between the separated groups of anaphase chromosomes. Cells were stained for DNA/chromosomes (DAPI, blue) and centrioles (γ-tubulin, green).
